# Antioxidant Activity of Butyl- and Phenylstannoxanes Derived from 2-, 3- and 4-Pyridinecarboxylic Acids

**DOI:** 10.3390/molecules15085445

**Published:** 2010-08-09

**Authors:** Alicia Corona-Bustamante, Juan Manuel Viveros-Paredes, Angelina Flores-Parra, Ana Lilia Peraza-Campos, Francisco J. Martínez-Martínez, María Teresa Sumaya-Martínez, Ángel Ramos-Organillo

**Affiliations:** 1 Facultad de Ciencias Químicas, Universidad de Colima, kilómetro 9 carretera Colima, Coquimatlán, Col. Mexico. C.P. 28400, Mexico; 2 Centro Universitario de Ciencias Exactas e Ingenierías, Universidad de Guadalajara, Laboratorio de Inmunofarmacología, Blvd. Marcelino García Barragán No. 1421, esq. Calzada Olímpica, C.P. 4430, Guadalajara, Jal., Mexico; 3 Departamento de Química, Centro de Investigación y de Estudios Avanzados-IPN. Apartado, Postal 14-740. Mexico 07000, D. F., Mexico; 4 Unidad Académica de Agricultura, Universidad Autónoma de Nayarit, Cd. de la Cultura "Amado Nervo", Boulevard Tepic-Xalisco s/n. C.P. 63190. Tepic, Nayarit, Mexico

**Keywords:** antioxidant activity, pyridinecarboxylate, organotin, DPPH, ferric-reducing

## Abstract

*In vitro* antioxidant activity for 12 stannoxanes derived from Ph_3_SnCl (compounds **1**-**3**), Ph_2_SnCl_2_ (compounds **4**-**6**), Bu_3_SnCl (compounds **7**-**9**), and Bu_2_SnCl_2_ (compounds **10**-**12**), was assayed qualitatively by the chromatographic profile with 1,1-diphenyl-2-picrylhydrazil (DPPH) method and by two quantitative methods: the DPPH radical scavenging activity and Ferric-Reducing Antioxidant Power (FRAP) assays. The results were compared with those obtained with the starting materials 2-pyridine- carboxylic acid (**I**), 3-pyridinecarboxylic acid (**II**) and 4-pyridinecarboxylic acid (**III**), as well as with standard compounds, such as vitamin C and vitamin E, respectively. The *in vitro* antiradical activity with DPPH of diphenyltin derivative **5** showed a very similar behavior to vitamin C at a 20 μg/mL concentration, whereas according to the FRAP method, compound **8** was better. This difference is due to the mechanism of the antioxidant process. The Structure-Activity Relationships (SAR) for both methods is also reported.

## 1. Introduction

Bioorganometallic chemistry is dedicated to the study of metallic complexes as well as their biological applications, with a view to designing new drugs offering better performance than those already known. Molecules have been designed for the treatment of cancer [1,2,3], Alzheimer’s [4], neurodegenerative diseases [5], as therapeutic and diagnosis agents [6], microbiocides [7,8], chelators [9], among others, where the principal characteristic is the presence of a metallic moiety which increases the biological activity compared to the free organic ligand. 

Involved in organometallic chemistry, we are interested in the structural and pharmacological activity of metallic complexes of group 14. These elements have shown great chemical versatility, as a result of their diverse electronegativities and their ability to increase their coordination spheres [10]. Germanium complexes are used in the fabrication of electronic components, chemotherapeutics, and as radioprotective agents [11], while complexes derived from heavier metals like tin and lead are relevant for their industrial applications as well as for their pharmacological and toxicological activities [11]. 

Applications of organotin(IV) compounds, especially those derived from carboxylate ligands, as bactericides [12,13,14,15,16], antitumoral [17,18,19], anti-inflammatory [20,21] and antifungal agents [13,15,16,22], as wood preservatives and as catalysts [23,24,25] and pesticides have been exhaustively studied to find the best performance depending on the ligand attached to the organometallic fragment, as well as the origin of the organotin moiety. 

This research is focused on the antioxidant activity of Sn(IV) compounds, to find out if there is an increased activity with the presence of the metallic center. Some authors have indicated that the antioxidant capacity of some flavonoids, like quercetin, is increased when it is complexed with a metallic moiety, for example Cu(II) [26], Cr(III) [27] or Co(II) [28]. There is little information however on a similar trend of Sn(IV) compounds; although there is information where authors refer that an equimolar mixture of the kaempeferol or quercetin (natural antioxidants) with diphenyltin dichloride (Ph_2_SnCl_2_) or triphenyltin chloride (Ph_3_SnCl) resulted in a higher antioxidant activity than that of kaempeferol or quercetin alone [29,30].

## 2. Results and Discussion

**Figure 1:**
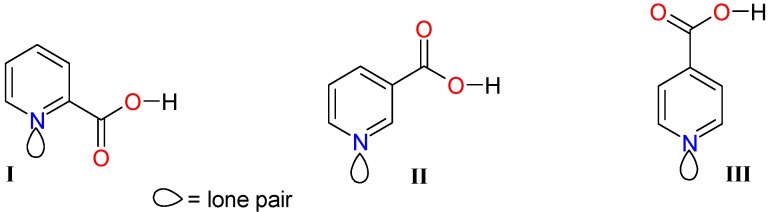
Ligands used to synthesize stannoxanes **1**-**12**.

Pyridinecarboxylic acid isomers **I**-**III** (Figure 1) are ligands with known biological activity [31,32,33,34,35], but no antioxidant activity is reported for any of them as we could corroborate running the DPPH and FRAP methods used in this work. The stannoxanes were synthesized as reported in the literature, all spectroscopic data are according to those reported for compounds **1**-**3** [36,37], **7**-**9** [38] and for **4**-**6** [36,39]. Here we have included the microanalyses as further evidence.

**Figure 2:**
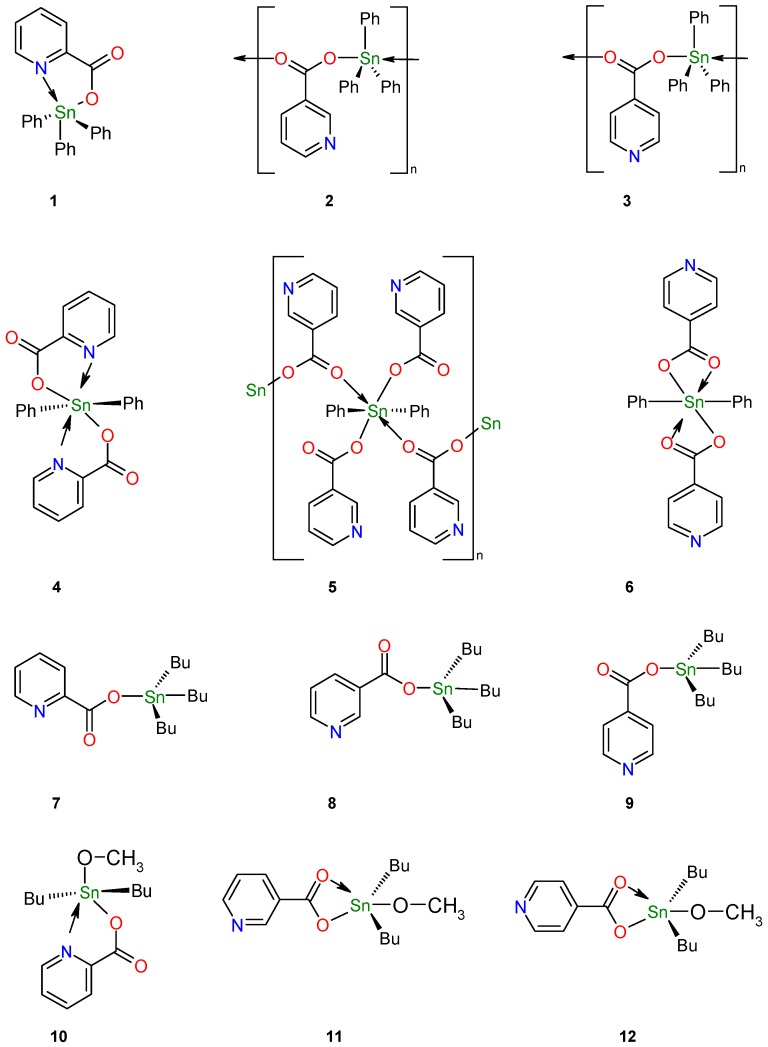
Structures for stannoxanes derived from 2-, 3- and 4-pyridinecarboxylic acids; pentacoordinated triphenylstannoxanes **1**-**3** [36,37]; hexacoordinated diphenylstannoxanes **4**-**6** [36,39]; tetracoordinated tributylstannoxanes **7**-**9** [38]; pentacoordinated dibutyl- stannoxanes **10**-**12**.

Compounds **10**-**12** were synthesized using a modified method reported by Gielen [40], the details and spectroscopic data for those compounds are summarized in the Experimental section and the structures are shown in Figure 2. 

Tin atom coordination number is important because the SAR analysis depends on it; this information allows us to compare the antioxidant activity of stannoxanes **1**-**12** (Figure 2) with other reported activities, such as antitumoral and bactericidal activities. In Figure 2 different stannoxane structures may be observed: the presence of N atom and the versatility of carboxylic group showed a variety of geometries such as tetrahedral (**7**-**9**), bipyramidal trigonal (**1**-**3** y **10**-**12**) and octahedral (**4**-**6**) in addition to the higher solubility in non-polar solvents provided by butyl rather than phenyl entities.

### 2.1. Thin Layer Chromatographic (TLC) profile modified with DPPH method

A very useful method to measure the antiradical activity of a compound is the one based on the use of the stable free radical DPPH, the electronic delocalization of this radical is responsible for its characteristic deep violet color. Compounds capable of donating a hydrogen atom will convert the DPPH radical into its reduced form, a neutral stable molecule, resulting in a color switch from deep violet to pale yellow [41,42], this method was selected because of its simplicity and worldwide acceptance, and the fact it enables us to compare results [43] with the ones already reported.

This assay was performed before the quantitative method to determine if stannoxanes were capable of stabilizing a radical molecule or not. No data concerning this kind of assays with stannoxanes could be found in the literature, so no comparison with other stannoxanes was possible. Nevertheless, there are reports concerning quantitative assays with metallic complexes of metals other than Sn(IV), which will also be discussed. 

**Figure 3:**
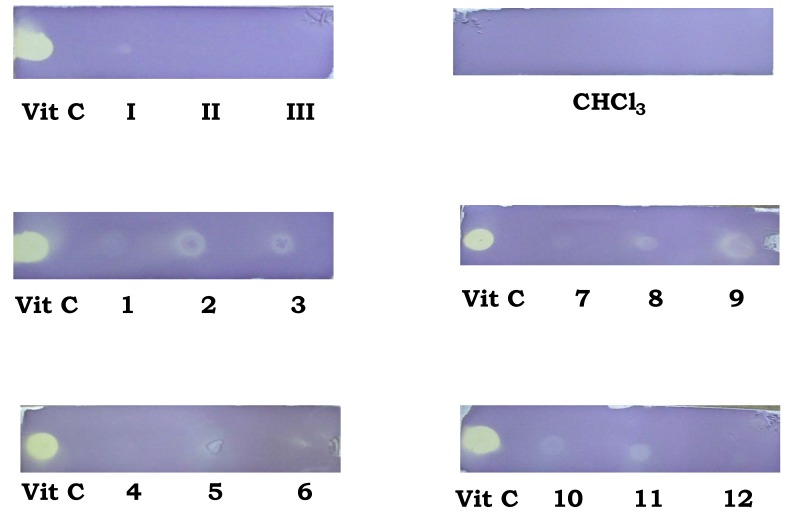
TLC fingerprints of stannoxanes *vs* vitamin C at 200 μg/mL in 0.2% methanolic DPPH solution, after 5–8 min.

Compounds **2**, **3, 5, 6, 8**, **9**, **10** and **11** presented discoloration on the application zone in the Thin Layer Chromatography (TLC) assay (Figure 3) which indicated that these compounds could have interesting results in the quantitative *in vitro* evaluation using the same radical. 

### 2.2.DPPH radical scavenging activity

**Figure 4:**
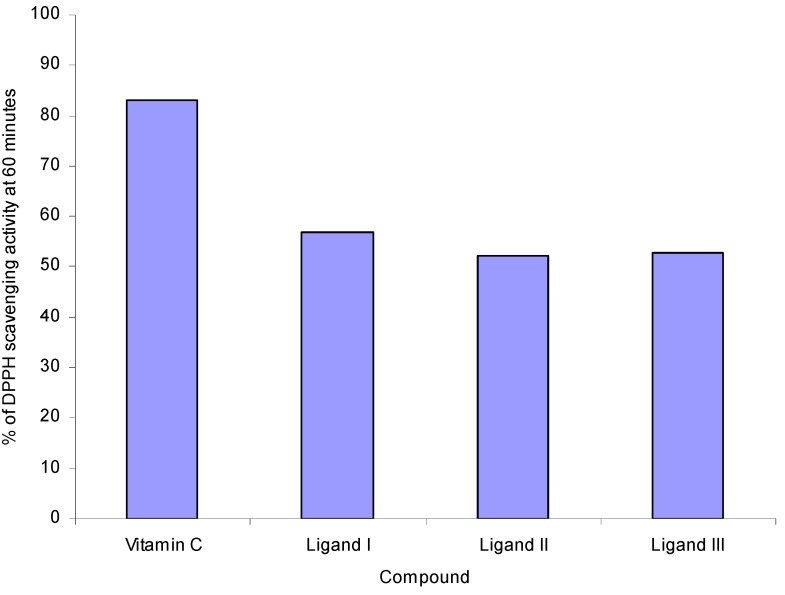
DPPH scavenging activity of ligands **I**-**III** *vs* vitamin C. Ligands **I**-**III** and vitamin C standard were analyzed at 20 μg/mL DMSO solutions at T = 60 min.

There are literature reports of metallic complexes where the ligand has antioxidant activity and it is expected that the metal moiety will increase its activity [26,29,44,45,46]. In this case, the three heterocarboxylic acid isomers show a moderated antioxidant activity even though, their use in diverse applications has been reported, for example, ligand **II** is an antihyperlipidemic drug and its widely used in patients with atherosclerosis [47]; also for ligand **III**, some authors refer its wide biological applications but these do not include antioxidant activity [48]. Ligands **I**-**III** (Figure 1), were tested for their antiradical activity at a concentration of 20 μg/mL. Figure 4 shows the antiradical activity of free ligands **I**-**III** at 60 min compared with vitamin C; the three isomers reach scarcely the 50% of antiradical activity. This information is consistent with that reported in a study on the inhibition of oxidation of Low Density Lipoproteins (LDL) where authors found that 2,6-pyridinedicarboxylic acid inhibited the oxidation of LDL, and no such activity on LDL was found for 2-pyridinecarboxylic acid (ligand **I**) [49]. 

Next the antiradical activity of the stannoxane complexes was tested at 60 min at a 20 μg/mL concentration in order to identify the molecule with best activity for each compound and be able to evaluate the structure-activity relationship [42,50]. 

**Figure 5:**
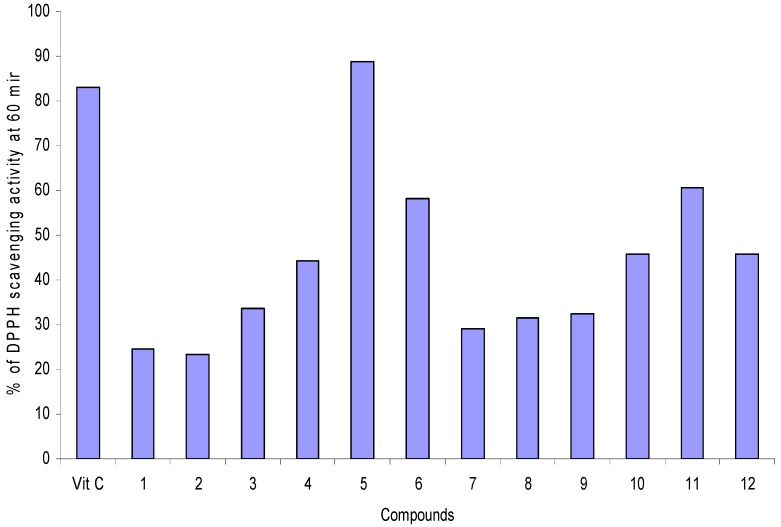
DPPH scavenging activity at T = 60 min. Stannoxanes and standard vitamin C were analyzed as 20 μg/mL DMSO solutions.

The evaluation of stannoxanes proceeded under the same conditions as for ligands **I**-**III**, and the DPPH scavenging activity results for stannoxanes **1**-**12**, showed that stannoxanes **5**, **6** and **11** (Figure 5) presented the best percentage of antiradical activity. 

The presence of the metallic moiety increased the antioxidant activity of the ligand because their proton donor capacity was enhanced. It is important to notice that stannoxane **5** had a very similar behavior to vitamin C. 

This is a promising result in the wide spectrum biological activity of organotin(IV) compounds; because this kind of compounds could also show a relevant antioxidant activity despite the fact of their implicit toxicity [51]. It seems that a diphenyltin moiety could participate in the antioxidative activity. Gabrielska *et al*. [29] found that the addition of Ph_2_SnCl_2_ to quercetin increased its antioxidant activity. In our research, ligand **II** did show a moderate antioxidant activity towards the DPPH stable free radical, but the complex derived from this ligand and Ph_2_SnCl_2_ (Figure 2, compound **5**) did show a high percentage of scavenging activity (Figure 5). This information confirms that the observed antioxidant activity is increased by the presence of a Sn(IV) metal center, as previously reported for other metals [26,44,45].

**Scheme 1:**
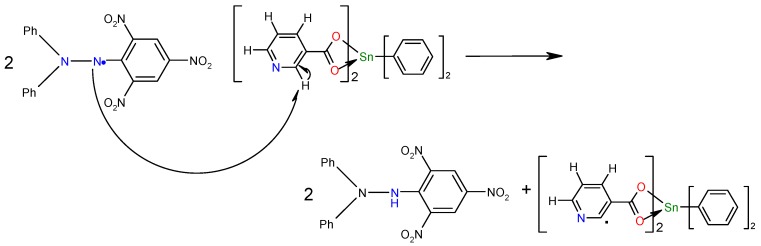
Proposed mechanism for DPPH scavenging activity for compound **5**.

The effect of stannoxanes **5**, **6** and **11** on DPPH radical scavenging could be due to their hydrogen donating ability. A possible exchanging mechanism is that in which a proton from the ligand is donated and the charge is stabilized through the entire complex, changing its charge delocalization capacity as shown in Scheme 1. The structure-activity relationship of the 12 stannoxanes (Figure 5) presents a behavior in which hexa- and pentacoordinated compounds (**4**-**6** and **10**-**12**) are the most capable for antiradical activity with DPPH. In the hexa- and pentacoordinated compounds the pyridine nuclei is uncovered in the structure because of the intramolecular coordination of the carboxylic group, except for compounds **4** and **10**, where the nitrogen of the pyridine nuclei is coordinating to the Sn(IV) center; for compounds **10**-**12** the presence of a O-CH_3_ group favors the proton donating ability. Compound **5** showed the best antiradical activity of the 12 stannoxanes.

### 2.3. Ferric-reducing antioxidant power assay *(FRAP)*

**Figure 6:**
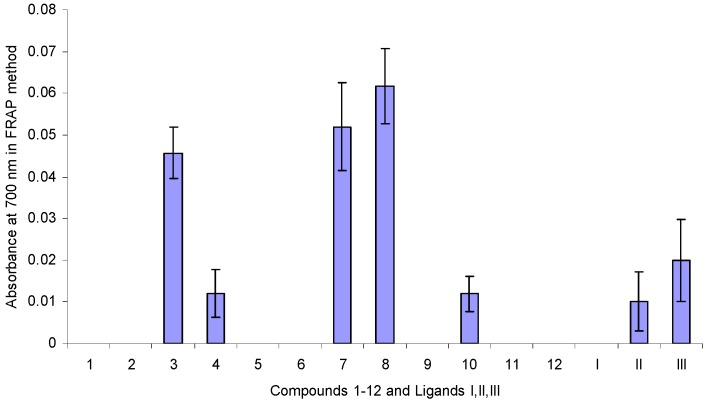
Iron(III) to Iron(II) reducing capacity for organic free ligands and stannoxanes at a 2 μg/mL concentration reported as absorbance formed at 700 nm.

We have screened antioxidant activity of the stannoxanes to evaluate their electron-donating activity [52] by the FRAP method, which is one of the most commonly used for this purpose. FRAP capacity was assayed for ligands **I**-**III** (Figure 1) and stannoxanes **1**-**12** (Figure 2) at a concentration of 2 μg/mL. Results are reported as absorbance at 700 nm (Figure 6). The 12 stannoxanes and ligands **I**-**III** did show significant Fe(III) reducing capacity (p < 0.05). Compound **8** showed the best reducing capacity among those derived from tributyltin. The final ranking was: **8 **> **7 **= **3 **> **III **> **II **= **4 **=**10**.

Analyzing their structures, their electron donating capacity can be explained by the presence of the free carboxylate group, as in the case of compounds **4** and **3** derived from diphenyltin and triphenyltin, respectively. We can argue that the electron-donating character of butyl group in **8** and **7**, and the electron-withdrawal of phenyl group in **4** and **3** are not determining their ferric-reducing power, in contrast to the coordination form of tin entity, because **8** and **7** are tetracoordinated and **4** and **3** are hexacoordinated molecules. 

**Scheme 2:**
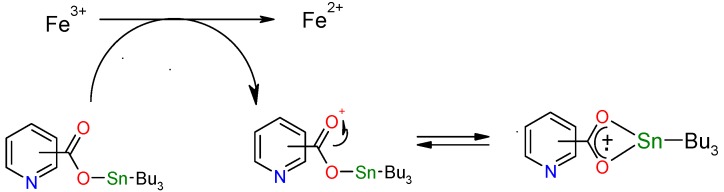
Proposed mechanism for FRAP method for tributyltin derivatives.

There are no data to compare these results with other metallic complexes; especially with organometallic compounds derived from Sn(IV). In this research, it is proposed a mechanism where an electron is donated in all analyzed complexes, the carboxylate group is exposed and the radical cation is delocalized through a four-member heterometallic cycle (Scheme 2). We continue working on this field to confirm this statement.

## 3. Experimental

### 3.1. General

For the synthesis of compounds **1**-**12**, all chemicals were reagent grade and were used without further purification. For the characterization of the compounds to compare results with those reported in the literature, melting points were measured by the capillary method in a Mel-Temp apparatus, and data are uncorrected. IR spectra were recorded on a Perkin Elmer FT-IR 1600 spectrophotometer using an ATR unit for solids in the 4,000–400 cm^-1^ range. The NMR spectra were obtained in CDCl_3_ and DMSO-d_6_ on Bruker 300 MHz or Jeol Eclipse 400 MHz spectrometers operations at 300.13185 MHz or 399.78219 MHz for ^1^H and75.47564 MHz or 100.52530 MHz for ^13^C, respectively, using TMS as internal reference, and Me_4_Sn for ^119^Sn (111.92607 MHz or 149.08124), respectively. Chemical shifts (δ) are reported in ppm [53]. For compounds **10** and **12** the ^119^Sn data was not recorded because of the insolubility of the mixture in DMSO-d_6_, and the partial solubility just let us obtain the ^1^H and ^13^C data. Antiradical activity with DPPH absorbance was measured with a Bio-Rad Benchmark microplate reader at 570 nm. Ferric-reducing antioxidant power assay absorbance was measured on a Perkin Elmer Lambda 2S UV/VIS spectrometer at 700 nm, the incubation of the samples was carried out at Pierce Reacti-Therm Heating Module and centrifugation of the samples was done at a Heraeus 3_S-R_ multifuge. 

### 3.2. Synthesis

Organotin compounds were synthesized following the method reported by Gielen *et al.* [40] with some modifications. The reactions were carried out in benzene solution for complexes **1**-**3** and **7**-**9** and in a mixture of toluene/methanol for complexes **4**-**6** and **10**-**12** to assure the synthesis and purity of compounds the ^1^H, IR spectra and melting point (m.p.) data were compared with those reported in literature; for compounds **1**-**3** [36,37], for **4**-**6** [36,39], and for **7**-**9** [38], these all were in agreement.

*Triphenyltin(IV) 2-pyridinecarboxylate* (**1**). White powder, yield 95%, IR (solid, υ): 1329 (C=O sym), 1675 (C=O asym), 1598 (C=C py), 1432 (C=N py), 532 (Sn-C), 436 (Sn-O); NMR (CDCl_3_): ^119^Sn: ‑26.3; ^1^H: H3 (8.39), H4 (7.98), H5 (7.53), H6 (8.61), H9 (7.55), H10 (7.21), H11 (7.21); Elem. anal.: [C_24_H_19_NO_2_Sn] Calc. C (61.06), H (4.6), N (2.97). Found C (61.01), H (4.12), N (2.84).

*Triphenyltin(IV) 3-pyridinecarboxylate* (**2**). White powder, yield 24%, IR (solid, υ): 1426 (C=O sym), 1650 (C=O asym), 1592 (C=C py), 1479 (C=N py), 509 (Sn-C), 492 (Sn-O); NMR (CDCl_3_): ^119^Sn: ‑107; ^1^H: H2 (9.2), H4 (7.3), H5 (8.3), H6 (8.6), H9 (7.79), H10 (7.48), H11 (7.46); Elem. anal.: [C_24_H_19_NO_2_Sn] Calc. C (61.06), H (4.6), N (2.97). Found. C (60.66), H (4.05), N (2.90).

*Triphenyltin(IV) 4-pyridinecarboxylate* (**3**). White powder, yield 86%, IR (solid, υ): 1344 (C=O sym), 1646 (C=O asym), 1543 (C=C py), 1473 (C=N py), 517 (Sn-C), 491 (Sn-O); NMR (CDCl_3_): ^119^Sn: -112; ^1^H: H2 (7.88), H3 (8.59), H5 (8.59), H6 (7.88), H9 (7.81), H10 (7.47), H11 (7.47); Elem. anal.: [C_24_H_19_NO_2_Sn] Calc. C (61.06), H (4.6), N (2.97). Found C (60.10), H (3.98), N (2.87).

*Diphenyltin(IV) 2-pyridinecarboxylate* (**4**). White powder, yield 31%, IR (solid, υ): 1326 (C=O sym), 1673 (C=O asym), 1595 (C=C py), 1461 (C=N py), 526 (Sn-C), 473 (Sn-O); NMR (CDCl_3_): ^119^Sn: ‑427; ^1^H: H3 (8.35), H4 (7.98), H5 (7.51), H6 (8.67), H9 (7.52), H10 (7.2), H11 (7.19); Elem. anal.: [C_24_H_18_N_2_O_4_Sn∙C_6_H_6_] Calc. C (60.54), H (4.06), N (4.71); Found C (59.18), H (4.85), N (3.97). 

*Diphenyltin(IV) 3-pyridinecarboxylate* (**5**). White powder, yield 23%, IR (solid, υ): 1360 (C=O sym), 1651 (C=O asym), 1585 (C=C py), 1467 (C=N py), 491 (Sn-C), 490 (Sn-O); NMR (DMSO-d_6_): ^119^Sn: -233.5; ^1^H: H2 (9.07), H4 (7.56), H5 (8.37), H6 (8.71), H9 (7.85), H10 (7.26), H11 (7.26); Elem. anal.: [C_24_H_18_N_2_O_4_Sn∙C_6_H_6_] Calc. C (60.54), H (4.06), N (4.71); Found C (59.98), H (4.62), N (4.50).

*Diphenyltin(IV) 4-pyridinecarboxylate* (**6**). White powder, yield 35%, IR (solid, υ): 1341 (C=O sym), 1641 (C=O asym), 1549 (C=C py), 1479 (C=N py), 534 (Sn-C), 492 (Sn-O); NMR (CDCl_3_): ^119^Sn: ‑233.5; ^1^H: H2 (7.58), H3 (8.33), H5 (8.33), H6 (7.58), H9 (7.73), H10 (7.14), H11 (7.14); Elem. anal.: [C_24_H_18_N_2_O_4_Sn∙C_6_H_6_] Calc. C (60.54), H (4.06), N (4.71); Found C (59.75), H (4.22), N (4.12).

*Tributyltin(IV) 2-pyridinecarboxylate* (**7**). Yellow liquid, yield >90%, IR (liquid, υ): 1395 (C=O sym), 1553 (C=O asym), 541 (Sn-C), 496 (Sn-O); NMR (CDCl_3_): ^119^Sn: +115.0; ^1^H: H3 (8.09), H4 (7.71), H5 (7.31), H6 (8.64), H8 (1.58), H9 (1.28), H10 (1.28), H11 (1.28); Elem. anal.: [C_18_H_31_NO_2_Sn] Calc. C (52.46), H (7.58), N (3.40). Found C (52.35), H (7.97), N (3.80).

*Tributyltin(IV) 3-pyridinecarboxylate* (**8**). Yellow liquid, yield >90%, IR (liquid, υ): 1344 (C=O sym), 1625 (C=O asym), 1460 (C=N py), 557 (Sn-C), 506 (Sn-O); NMR (CDCl_3_): ^119^Sn: +117.5; ^1^H: H2 (9.1), H4 (7.2), H5 (8.2), H6 (8.6), H8 (1.63), H9 (1.34), H10 (1.34), H11 (1.34); Elem. anal.: [C_18_H_31_NO_2_Sn] Calc. C (52.46), H (7.58), N (3.40). Found C (52.13), H (8.12), N (3.80).

*Tributyltin(IV) 4-pyridinecarboxylate* (**9**). White powder, yield 23%, IR (solid, υ): 1348 (C=O sym), 1646 (C=O asym), 1603 (C=C py), 1460 (C=N py), 521 (Sn-C), 492 (Sn-O); NMR (CDCl_3_): ^119^Sn: +116.8; ^1^H: H2 (8.62), H3 (7.81), H5 (7.81), H6 (8.62), H8 (1.58), H9 (1.29), H10 (1.29), H11 (1.31); Elem. anal.: [C_18_H_31_NO_2_Sn] Calc. C (52.46), H (7.58), N (3.40). Found C (52.59), H (8.09), N (3.83).

*Dibutyltin(IV) methoxy-2-pyridinecarboxylate [(C_6_H_4_NO_2_)(CH_3_O)Sn(C_4_H_9_)_2_]* (**10**). General procedure: a solution of **I** (0.5 g, 4.06 mmol) and Et_3_N (0.6 mL, 0.436 g, 4.30 mmol) in toluene (30 mL) and ethanol (30 mL) was mixed with continuous stirring with another solution of dibutyltin chloride (1.39 g, 4.57 mmol) and Et_3_N (0.6 mL, 0.436 g, 4.30 mmol) in toluene (30 mL) and ethanol (10 mL). The resulting mixture was heated by 8 h with continuous stirring. At the end of the reaction, the mixture was filtered off and the solvent was evaporated at low pressure, the resulting product was washed with chloroform to give a white powder, yield 90%, m.p. 146–148 ºC, IR (solid, υ): 1346 (C=O sym), 1664 (C=O asym), 1620 (C=C py), 1457 (C=N py), 541 (Sn-C), 468 (Sn-O); NMR (DMSO-d_6_): ^119^Sn: not observed; ^1^H: H3 (8.38), H4 (8.02), H5 (7.60), H6 (8.98), H8 (1.30), H9 (1.12), H10 (1.12), H11 (1.30), O-Me (3.01); ^13^C: C2 (147.5), C3 (127.6), C4 (140.5), C5 (125.6), C6 (146), C7 (166.7), C8 (28.8), C9 (27.6), C10 (26.4), C11 (13.6), O-Me (46.5); Elem. anal.: [C_15_H_25_NO_3_Sn] Calc. C (46.67), H (6.53), N (3.63). Found C (45.91), H (7.06), N (3.91).

*Dibutyltin(IV)-methoxy-3-pyridinecarboxylate [(C_6_H_4_NO_2_)(CH_3_O)Sn(C_4_H_9_)_2_]* (**11**). Using the same method reported for **10**, **II **(0.5 g, 4.06 mmol) and Et_3_N (0.6 mL, 0.436 g, 4.30 mmol) in a solution of toluene (30 mL) and ethanol (10 mL) was mixed with dibutyltin dichloride (1.39 g, 4.57 mmol) and Et_3_N (0.6 mL, 0.436 g, 4.30 mmol) in a mixture of toluene (30 mL) and ethanol (10 mL). Yield 36%, m.p. 188–190 ºC, IR (solid, υ): 1395 (C=O sym), 1599 (C=O asym), 1551 (C=C py), 1459 (C=N py), 543 (Sn-C), 438 (Sn-O); NMR (CDCl_3_): ^119^Sn: -211; ^1^H: H2 (9.09), H4 (7.32), H5 (8.19), H6 (8.63), H8 (1.59), H9 (1.25), H10 (1.25), H11(0.755), O-Me (3.03); ^13^C: C2 (151.3), C3 (130), C4 (137.8), C5 (123.6), C6 (153), C7 (171.7), C8 (27.7), C9 (27.0), C10 (27.0), C11 (13.9), O-Me (45.8); Elem. anal. [C_15_H_25_NO_3_Sn] Calc. C (46.67), H (6.53), N (3.63). Found C (45.41), H (7.15), N (3.83).

*Dibutyltin-methoxy-4-pyridinecarboxylate [(C_6_H_4_NO_2_)(CH_3_O)Sn(C_4_H_9_)_2_]* (**12**). Using the same method reported for **10**, **III **(0.5 g (4.06 mmol) of and 0.6 mL (0.436 g, 4.30 mmol) of Et_3_N in a mixture of 30 mL of toluene and 10 mL of ethanol, was mixture with 1.39 g (4.57 mmol) of dibutyltin dichloride and 0.6 mL (0.436 g, 4.30 mmol) of Et_3_N in solution of 30 mL of toluene and 10 mL of ethanol, the product was a yellow liquid, yield >90%, IR (solid, υ): 1394 (C=O sym), 1598 (C=O asym), 1549 (C=C py), 1468 (C=N py), 491 (Sn-C), 437 (Sn-O); NMR (CDCl_3_): ^119^Sn: not observed; ^1^H: H2 (8.582), H3 (7.676), H5 (7.676), H6 (8.582), H8 (1.656), H9 (1.34), H10 (1.34), H11 (0.957), O-Me (3.05); ^13^C: C2 (150.3), C3 (123.7), C4 (141.7), C5 (123.1), C6 (150.3), C7 (170.9), C8 (28.2), C9 (27.7), C10 (27.3), C11 (13.9), O-Me (46.2); Elem. anal. [C_15_H_25_NO_3_Sn] Calc. C (46.67), H (6.53), N (3.63). Found C (45.41), H (7.33), N (3.99).

### 3.3. Thin Layer Chromatographic (TLC) profile modified with DPPH method

The methodology reported by Conforti *et al.* [54] was applied with modifications, due to the fact that characteristically the organometallic fragment of stannoxanes can bind to the TLC layer and possibly leave free the organic fragment; in this case the chromatographic layer was not developed in any solvent, so, the stannoxanes were applied in the TLC layer and left to dry under room conditions. A 200 μg/mL CHCl_3 _solution of each stannoxane **1**-**12** as well as the ligands **I**-**III** and a 0.2% DPPH methanolic solution were prepared. A standard of vitamin C at 200 μg/mL concentration was also prepared. After that they were applied to TLC layers as spots of approximately 10 μL of each compound as well as the vitamin C standard and allowed to dry under room conditions for 30 min. Then the layers were submerged in 0.2% DPPH solution. Once the reaction of the free stable radical was finished (about 5-8 min) a discoloration was shown in the spot applied for any compound showing antioxidant activity. 

### 3.4. Antiradical activity measurement with DPPH

The antiradical activity of the stannoxanes derived from pyridinecarboxylic acids was carried out using the method proposed by Blois [55]; dilutions in DMSO solvent for the 12 stannoxanes and ligands I-III at 20 μg/mL, and a 0.01 mM methanolic solution of DPPH stable radical were prepared.

Dilutions of the stannoxanes and ligands were applied in a 96-well microplate and a blank without sample was also prepared. After the application of all the compounds the 0.01 mM DPPH solution was added and absorbance at time zero (T = 0) was immediately measured using a UV wavelength of 570 nm. Measurements were performed every 5 min until 30 min was completed and every 15 min until 90 min were completed. Vitamin C was used as standard molecule at a 20 μg/mL concentration. 

Antiradical activity evaluation for stannoxanes **1**-**12** and ligands **I**-**III** was measured in terms of absorbance decrease at 570 nm of DPPH methanolic solution produced by the effect of each stannoxane or ligand as a result of their ability to donate a hydrogen giving place to the reduced form of DPPH radical (pale yellow solution). The amount of DPPH reduced form was determined in every established period of time using the following equation: [(Ao-Ae)/Ao]*100, where **Ao** corresponds to the absorbance of sample without stannoxane and **Ae** corresponds to the absorbance of sample with stannoxane.

In this article, we decided to do an analysis of antiradical activity at 60 min (T = 60 min) at 20 μg/mL concentration in order to present the best activity for each compound and ligand and be able to evaluate the structure-activity relationships [42,50]. 

### 3.5. Ferric-reducing antioxidant power assay (FRAP)

The ability of the stannoxanes **1**-**12** as well as the ligands **I**-**III** to reduce iron(III) was carried out by the method reported by Hinneburg *et al.* [52], scaling it to a micro method as follows: a 2 μg/mL solution of each stannoxane and ligand in DMSO was prepared. In an assay tube 400 μL of stannoxane solution, 1000 μL of PBS buffer, and 1000 μL of K_3_Fe(CN)_6_, were added and this mixture was incubated for 30 min at 50 ºC, after this, 10% trichloroacetic acid solution (1000 μL) was added to the sample and this mixture was stirred for one minute and centrifuged at 1,500 rpm for 10 min; after the centrifugation a 1000 μL aliquot of the supernatant was taken and transferred to a clean assay tube where 0.1% FeCl_3_ solution (200 μL) was added. The absorbance of this mixture was recorded at 700 nm in an UV-spectrophotometer, the values are presented as means ±SD, n = 3.

## 4. Conclusions

Two methods were used to determine the antioxidant power of stannoxanes; the proton and the electron donating ability were determined, and the results show that the bound and free pyridine and carboxylic nuclei were determinant for this, and the butyl- and phenyl groups play an important role in tin(IV) coordination, because of their electron-donating and electron-withdrawing capabilities. The results show that six of the twelve stannoxanes evaluated are good radical scavengers, compounds **5** > **6** = **11** in the DPPH quantitative assay and compounds **8** > **7** = **3** in the FRAP method. The most promising compound, in the context of this work, is compound **5**. For DPPH, diphenyltin and dibutyltin (hexa- and pentacoordinated respectively) were the best, being the most active diphenyltin-*bis*-(3-pyridinecarboxylate) (compound **5**) the most active. In the FRAP method tributyltin-3-pyridinecarboxylate (compound **8**, tetracoordinated) was the most active derivative. 
